# Adsorption of protein antigen to the cationic liposome adjuvant CAF®01 is required for induction of Th1 and Th17 responses but not for antibody induction

**DOI:** 10.1016/j.ejpb.2021.05.020

**Published:** 2021-08

**Authors:** Katharina Wørzner, Jóhanna Hvannastein, Signe Tandrup Schmidt, Camilla Foged, Ida Rosenkrands, Gabriel Kristian Pedersen, Dennis Christensen

**Affiliations:** a Statens Serum Institut, Department of Infectious Disease Immunology, Artillerivej 5, 2300 Copenhagen S, Denmark; b Department of Pharmacy, Faculty of Health and Medical Sciences, University of Copenhagen, Universitetsparken 2, DK-2100 Copenhagen Ø, Denmark

**Keywords:** CAF01, Adjuvant, T-cell response, Depot, Adsorption, Succinylation

## Abstract

The degree of antigen adsorption to adjuvants in subunit vaccines may significantly influence the immune responses they induce upon vaccination. Commonly used approaches for studying how the level of adsorption affects the induction of antigen-specific immune responses include (i) using adjuvants with different abilities to adsorb antigens, and (ii) comparing different antigens selected based on their ability to adsorb to the adjuvant. A weakness of these approaches is that not only the antigen adsorption level is varied, but also other important functional factors such as adjuvant composition and/or the B/T cell epitopes, which may affect immunogenicity. Hence, we investigated how changing the adsorption capabilities of a single antigen to an adjuvant influenced the vaccine-induced immune responses. The model antigen lysozyme, which displays a positive net charge at physiological pH due to an isoelectric point (pI) of 11, was succinylated to different extents, resulting in a reduction of the pI value to 4.4–5.9, depending on the degree of succinylation. A pronounced inverse correlation was found between the pI value of the succinylated lysozyme analogues and the degree of adsorption to a cationic liposomal adjuvant consisting of dimethyldioctadecylammonium bromide (DDA) and trehalose dibehenate (TDB) (CAF®01). Furthermore, increased adsorption to this adjuvant correlated directly with the magnitude of lysozyme-specific Th1/Th17 immune responses induced by the vaccine in mice, while there was an inverse correlation with antibody induction. However, high lysozyme-specific antibody titers were induced with an increased antigen dose, even upon vaccination with a strongly adsorbed succinylated lysozyme analogue. Hence, these data illustrate that the degree of lysozyme adsorption to CAF®01 strongly affects the quality of the resulting immune responses.

## Graphical abstract

Non-adsorbed antigen administered with the depot-forming, cationic adjuvant formulation (CAF®01) results in low T cell responses, while adsorbed antigen increases T cell responses. Especially Th17 responses benefit from high adsorption. Abstract created with biorender.com.

**Figure:**
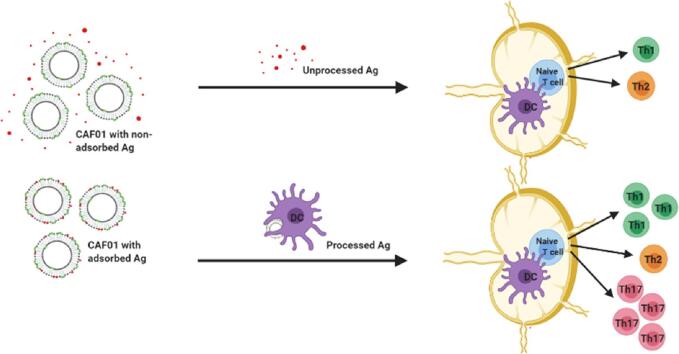

## 1 Introduction

Peptide- and protein-based subunit technologies are often used in novel vaccine candidates due to the high pathogen specificity and reduced adverse effects compared to the traditional whole-cell vaccines. However, the intrinsic low immunogenicity of most peptides and proteins necessitates the use of adjuvants to induce sufficient immune responses for achieving protective immunity.

The importance of (i) antigen (Ag) adsorption to adjuvants and (ii) vaccine depot formation, facilitated by the adjuvant, on the elicited immune response have been disputed ever since the first studies with alum-precipitated diphtheria toxoid a century ago [1], [2]. In 1931, Glenny and coworkers suggested that the increased antibody titers observed for alum-adjuvanted vaccines can be attributed to the slow clearance of alum-precipitated Ag from the site of injection (SOI), as compared to the clearance of non-adjuvanted, soluble Ag alone [3]. Later studies showed that a certain degree of Ag retention at the SOI is crucial for adequate activation of the immune system [4], [5], [6]. Hence, the adjuvant mechanism of aluminum-based adjuvants is attributed to the ability to adsorb Ags and form a depot at the SOI from which the Ag is slowly released, eventually potentiating antibody responses [7].

Many studies have investigated how Ag adsorption to different adjuvants and their depot formation upon injection affects humoral immune responses [8], [9], [10]. However, little is known about how Ag kinetics and dynamics influence T-cell responses. One study showed that subcutaneous (s.c.) injection of non-adjuvanted, soluble Ag results in rapid Ag translocation to the draining lymph nodes (LNs) where the Ag was processed by LN-resident dendritic cells (DCs) and presented to naïve T cells [5]. However, Ag presentation alone is not sufficient for the induction of effector T cells, which require costimulatory signals, preferably mediated by the Ag-presenting DCs migrating from the SOI. In this respect, co-localization of the Ag and the adjuvant is important [11]. Duration of Ag presentation is another factor suggested to be important for strong T cell induction [12] and studies have shown that at least partial retention of Ag at the SOI is essential for inducing strong effector T cell responses [5].

Depot-forming adjuvants, such as aluminum salts and cationic liposomes, function by retaining the Ag at the SOI. In these cases it is the physicochemical characteristics of the Ags and adjuvants that determine whether the vaccine form an Ag depot after injection [13], [14]. Hence, the physicochemical properties of adjuvants and Ags (size, surface charge and hydrophobicity) play a key role in the modulation of vaccine-induced immune responses [15], [16], [17]. In a recent study, the immune profiles of lead vaccine adjuvant candidates were compared with different Ags, *i.e.* (i) the aluminum-based adjuvant Alhydrogel®, (ii) the squalene-based oil-in-water emulsion MF59™, and (iii) the cationic liposome-based adjuvant CAF®01 composed of dimethyldioctadecylammonium bromide (DDA) and trehalose dibehenate (TDB). For all Ags, Alhydrogel® primarily increases antibody titers, MF59™ induces strong antibody and IL-5 responses, and CAF®01 shows a mixed Th1 and Th17 response [18]. We recently demonstrated that adjuvants influence the kinetics of antibody responses in different ways [10]. In particular adjuvants inducing a depot at the SOI (Alhydrogel® and CAF®01) resulted in delayed, but higher magnitude responses than a non-depot inducing adjuvant (MF59™-like squalene emulsion AddaVax™ (SE)). This suggests that sustained release of Ag, *e.g.* from depot-forming adjuvants, limits the dose of Ag available for B cells in the lymph nodes, which reduces early B cell activation and antibody induction [10].

In contrast to the aluminum- and squalene emulsion-based adjuvants, CAF®01 induces strong Th1/Th17-cell responses [19], which have been suggested to be dependent on the formation of an Ag depot at the SOI and/or the co-delivery of Ag and adjuvant to antigen-presenting cells (APCs) [11], [15], [16]. This is supported by data showing that a fluid phase analogue of CAF®01, *i.e.* dimethyldioleoylammonium (DODA)/TDB liposomes, which does not form a depot with the Ag at the SOI, mediates significantly lower T-cell responses than CAF®01 [15]. Non-adsorbed Ag administered side-by-side with CAF®01 and draining to the same LN, did also induce lower Th1 and Th17 responses compared to CAF®01-adsorbed Ag, but did not affect Th2 and antibody responses [11]. Finally, comparing CAF®01 with other cationic liposomes based on 3β-[N-(N′,N′-dimethylaminoethane)carbomyl] cholesterol (DC-Chol) or 1,2-dioleoyl-3-trimethylammonium propane (DOTAP), incorporating the same immunostimulator (TDB), showed that CAF®01 and the DC-Chol-based adjuvant displayed similar pharmacokinetics and biodistribution, with 40% of the dose remaining at the SOI two weeks after immunization. In contrast, the fluid phase DOTAP-based liposomes drained rapidly from the SOI, which resulted in a higher vaccine dose fraction in the draining LNs, but lower Th1 responses, compared to CAF®01 and DC-Chol [17].

While Ag depot formation at the SOI appears to be important for T cell induction, a weakness of the approaches used in the abovementioned studies is that important experimental factors, *e.g.* the Ag, the vaccine delivery system, and/or immunization method, are not kept constant; hence, these factors may also influence immunogenicity [11], [15], [20]. In the present study, a different approach was adopted to investigate the effects of Ag adsorption to CAF®01 on immunogenicity. Since Ag adsorption to CAF®01 is primarily driven by attractive electrostatic interactions [16], [20], the degree of Ag adsorption was modulated by changing the isoelectric point (pI) of the model Ag lysozyme (LYS) by succinylation [21]. LYS, displaying a positive net charge at physiological pH due to a pI of 11, was succinylated to various degrees resulting in a reduction of the pI value to 4.4, 5.0 and 5.9. We found that reducing the pI of LYS increased adsorption to CAF®01, and that immunizing with the resulting succinylated LYS analogues adjuvanted with CAF®01 boosts Ag-specific Th1/Th17 responses compared to unmodified LYS. Partial but not complete binding furthermore increased the Th2 responses.

## 2 Materials and methods

### 2.1 Materials

Chicken egg white LYS (>90%), succinic anhydride (≥99%), dimethyl sulfoxide (DMSO), chloroform and bovine serum albumin (BSA) were purchased from Sigma-Aldrich (St Louis, MA, USA). DDA and TDB were purchased from NCK A/S (Farum, Denmark). Disodium hydrogen phosphate dihydrate (Na_2_HPO_4_·2H_2_O), sodium dihydrogen phosphate monohydrate (NaH_2_PO_4_·H_2_O), sodium hydroxide pellets (NaOH), Tween® 20 and methanol were obtained from Merck KGaA (Darmstadt, Germany). Gibco phosphate-buffered saline (PBS) was purchased from Life Technologies Limited (Paisley, UK).

### 2.2 Succinylation of lysozyme

Succinylation of LYS was performed essentially as reported previously [22] with minor alterations. The succinylation reaction was conducted at four different theoretical molar ratios of succinic anhydride to LYS, *i.e.* 100, 10, 5 and 0.5, respectively, and the resulting LYS analogues are denoted LYS-100, LYS-10, LYS-5 and LYS-0.5. Unmodified lysozyme is simply denoted LYS. LYS was dissolved in sodium phosphate buffer (100 mM, pH 8) to a concentration of 20 mg/ml. Depending on the desired molar ratio, succinic anhydride was dissolved in 250 (LYS-10 (70 mg/ml), LYS-5 and LYS-0.5 (35 mg/ml)) or 500 (LYS-100 (340 mg/ml)) µl DMSO, respectively. The succinic anhydride solutions was added to the protein solutions (12.5 ml) in small aliquots over a period of 60 min under constant stirring. For the LYS-0.5, only 25 µl succinic anhydride and DMSO solution (35 mg/ml) was added over a period of 10 min, resulting in a succinic anhydride to lysozyme molar ratio of 0.5. The pH was maintained at 8–9 by adjusting the pH with 0.1 M NaOH solution. The reaction was allowed to proceed for 30 min at 4 °C. The LYS analogues were dialyzed for two days against deionized water using a volume corresponding to at least 200 times the sample volume in Slide-A-Lyzer™ Dialysis Cassettes (12–30 ml, Thermo Fisher Scientific, Waltham, MA, USA). The analogues were filtered through 0.2 nm Minisart® filters (Sartorius Stedim Biotech Göttingen, Germany). The final protein concentration was determined by UV spectroscopy at 280 nm using a NanoDrop™ 2000 spectrophotometer (Thermo Fisher Scientific).

The theoretical pI values of the succinylated LYS analogues were estimated from the amino acid sequence of hen egg white lysozyme [23]. The pI values were calculated using the online ProtParam tool program [24] applying a molar extinction coefficient, ε, of 37,370 M^−1^ cm^−1^ (A_0.1%_ = 2.653 mg ml^−1^ cm^−1^), by replacing lysine (K) and tyrosine residues (Y) with glutamate (E) residues (Supplementary Fig. 1). ProtParam does not allow for replacement of the N-terminal amine group, hence succinylation of this group was not accounted for in the calculations.

### 2.3 Gel electrophoresis

Gel electrophoresis [25–192 mM Tris-Glycine native polyacrylamide gel electrophoresis (PAGE)] was performed to investigate the shift in the charge-based mobility of the LYS analogue mixtures compared to unmodified LYS. Gel electrophoresis was run with 4–15% Mini-PROTEAN® TGX™ Precast Gels with 12 wells in a Mini-PROTEAN® Tetra Vertical Electrophoresis Cell using premixed electrophoresis buffer containing 25 mM Tris, 192 mM glycine, pH 8.3 (all from BioRad, Hercules, CA, USA). The protein samples were diluted 1:1 (v/v) in a non-SDS sample buffer consisting of 62.5 mM tris-HCl, 10% glycerol and bromophenol blue and approximately 2 µg protein was loaded into each well. The gel was run at 300 V for approximately 20 min followed by staining using Coomassie brilliant blue R-350 solution (GE Healthcare, Chicago, IL USA) for 1 h and destained with water for 3 × 5 min. Identically loaded gels were run with both normal (negative-to-positive) and reverse (positive-to-negative) positioning of the electrodes, the latter to verify presence of unmodified LYS and positively charged LYS analogues at pH > 8.3.

### 2.4 Isoelectric focusing

The pI value of the succinylated LYS analogues were determined by isoelectric focusing (IEF) using Novex™ pH 3–10 IEF Protein Gels, 1.0 mm, 10 wells, and the XCell SureLock™ Mini Gel Tank. The anode and cathode buffers were purchased from Thermo Fischer Scientific as a part of the Novex™ pH 3–10 IEF Buffer Kit and cooled to 4 °C before use. The samples were mixed with sample buffer (1:1, v/v) at a concentration leading to minimum 2 µg protein on the gel. The used marker was IEF Marker 3–10, SERVA Liquid Mix (ThermoFisher Scientific). The gel was run at 100 V for 1 h, followed by 200 V for 1 h and 500 V for 30 min. The gel was fixed with a 12% (v/v) trichloroacetic acid solution (TCA 20%, Carl Roth, Karlsruhe, Germany) for 30 min followed by staining using Bio-Safe™ Coomassie brilliant blue G-250 (GE Healthcare) solution for 1 h. The gel was destained in water for 3 × 5 min.

### 2.5 Matrix-assisted laser desorption/ionization mass spectrometry (MALDI-MS)

Mass spectrometry (MS), using the matrix assisted laser desorption time-of-flight (MALDI-TOF) method, was performed to determine the primary structure of LYS and the modified LYS analogues. MALDI-MS was performed by Alphalyse A/S (Odense, Denmark). The proteins were purified using a Millipore C18 ziptip (Merck Millipore, Darmstadt, Germany). Each purified sample was mixed with 2,5-dihydroxyacetonphonone/diammonium hydrogen citrate (DHAP/DAHC) matrix and spotted onto a Big Anchor target from Bruker (Bremen, Germany). A laser beam (Nd: YAG SmartBeam laser: 355 nm, 2,000 Hz repetition rate) was used to desorb the matrix crystals and the molecules were ionized in the gas phase. The mass spectra were acquired using an Autoflex Speed MALDI TOF spectrometer (Bruker).

### 2.6 Circular dichroism (CD)

Far UV CD was performed using a Chirascan™ spectrophotometer (Applied Photophysics, Leatherhead, UK) to evaluate the effect of succinylation on the secondary structure of lysozyme. The measurements were conducted at 25 °C in a 0.1 mm quartz cell (Starna®, Essex, UK). The samples were diluted in sodium phosphate buffer (100 mM, pH 8) to a protein concentration of 0.2 mg/ml. All spectra were recorded from 190 to 260 nm. A step size of 1.0 nm, a fixed bandwidth of 1.0 nm and a constant integration time of 1 s per point were used. All generated spectra are an average of three scans. The spectra were background-corrected automatically and the buffer scans were subtracted manually. Results are displayed as molar ellipticity (*θ*) on a per residue basis. The shown spectra have not been smoothed.

### 2.7 Hydrodynamic diameter

The average number-weighted hydrodynamic diameter of the succinylated lysozyme analogues was measured by dynamic light scattering (DLS) by using the photon correlation spectroscopy technique. The measurements were performed using a Zetasizer Nano ZS (Malvern Instruments, Worcestershire, UK) at 25 °C equipped with a 633 nm laser and a 173° detection optics. The sizes were measured in triplicate in micro cuvettes (Malvern Panalytical Ltd, Worcestershire, UK) at a protein concentration of 0.1 mg/ml in sodium phosphate buffer (100 mM, pH 8). For viscosity and refractive index, the values of water were used. Malvern Zetasizer v.7.12 software (Malvern Instruments) was used for data acquisition and analysis.

### 2.8 Preparation of adjuvant

CAF®01 was prepared using the lipid film hydration method as described previously [19]. DDA and TDB were weighed individually and dissolved in chloroform/methanol 9:1 (v/v). The two components were mixed at a mass ratio of 5:1, corresponding to a molar ratio of 89:11. The resulting solution was placed under nitrogen flow for approximately 1 h to evaporate the organic solvents, and the resulting lipid film was dried overnight to remove residual organic solvent. The vial was stored at −18 °C. The lipid film was rehydrated with 10 mM Tris-HCl buffer supplemented with 9% (w/w) trehalose (pH = 7.4) (Sigma-Aldrich) to a final theoretical lipid concentration of 1.5 mg/ml. Hydration was performed at 60 °C for 15 min with simultaneous homogenization at 26,000 rpm using a 6F SilentCrusher (Biohit, Helsinki, Finland).

### 2.9 Adsorption studies

Adsorptions studies were performed as described previously [20]. The samples included either unmodified LYS or one of the succinylated LYS analogues at different concentrations mixed with CAF®01. The samples were prepared by diluting the Ags in 10 mM Tris-HCl buffer + 9% trehalose (pH = 7.4) (Sigma-Aldrich). CAF®01 was added to the solution (1:1, v/v) and mixed by vortexing for 10 s with 10 min intervals for 30 min. Samples without adjuvant, Tris buffer and CAF®01 alone, respectively, were used as controls. The samples were centrifuged using an Optimal™ MAX-XP ultra centrifuge (Beckman Coulter, Ramcon, Copenhagen, Denmark) at 135,700g for 30 min. The supernatants were collected immediately after centrifugation, and the Ag-concentration was determined using the bicinchoninic acid (BCA) assay kit (Thermo Fisher Scientific). The absorbance was measured using a TECAN Sunrise™ enzyme-linked immunosorbent assay (ELISA) reader (Tecan Trading, Männedorf, Switzerland) at 570 nm. The Magellan™ software v6.6 (TECAN) was used for data acquisition. The amount of Ag adsorbed to the adjuvant was calculated by subtracting the amount of protein remaining in the supernatant after centrifugation from the total amount of added Ag measured in the samples without adjuvant. The results are given as the amount of lysozyme adsorbed/lipid (g/g), where the lipid is the amount of DDA in one mouse injection dose. In addition, the collected supernatants were analyzed by IEF to verify the Ags not complexed with CAF®01.

### 2.10 Preparation of vaccines

For the first *in vivo* experiment, vaccines containing unmodified LYS, modified LYS-5 or modified LYS-100 were prepared by mixing Ag with CAF®01 as previously described [19] at a dose of 5 µg protein and 300 µg CAF®01. For the second experiment, unmodified LYS and modified LYS-100 were included, and the vaccines were prepared at a dose of 5 µg protein. In addition, two higher doses of 20 and 40 µg of LYS-100 were tested. The vaccines were prepared under sterile conditions at a dose volume of 200 µl. The proteins were first diluted in 10 mM Tris buffer supplemented with 9% (w/v) trehalose (pH = 7.4) (Sigma-Aldrich) and 100 µl/dose of CAF®01 or buffer was subsequently added to the solution. The vaccines were mixed by vortexing for 10 s every 10th min for 30 min immediately before immunization.

### 2.11 Immunization

Female BALB/c mice (ENVIGO, Huntington, UK), aged 6–8 weeks, were kept at the experimental animal facilities at Statens Serum Institut and handled by authorized personnel only. All experimental work was conducted in accordance with the regulations of the Danish Ministry of Justice and the Danish National Experiment Inspectorate under permit 2017–15-0201–01363 and in compliance with the European Community Directive 86/609 for the care and use of laboratory animals. The mice were allowed free access to food and water. Two separate animal experiments were conducted. In the first experiment, mice (five or eight/group) were immunized s.c. three times with two-weeks intervals with 200 µl 5 µg Ag/dose at the base of the tail with (i) unmodified LYS, (ii) unmodified LYS adjuvanted with CAF®01, (iii) LYS-5 adjuvanted with CAF®01 and (iv) LYS-100 adjuvanted with CAF®01, respectively. Blood and spleens were harvested three weeks after the last immunization. In the subsequent experiment, 16 mice per group were immunized s.c. three times with two-weeks intervals with 200 µl at the base of the tail with (i) unmodified LYS (5 µg/dose), (ii) unmodified LYS (5 µg/dose) adjuvanted with CAF®01, (iii) LYS-100 (5 µg/dose) adjuvanted with CAF®01, (iv) LYS-100 (20 µg/dose) adjuvanted with CAF®01 and (v) LYS-100 (40 µg/dose) adjuvanted with CAF®01, respectively. Blood was drawn from four animals per group at 2, 4, 6 and 8 weeks after the last immunization.

### 2.12 Sample collection and cell preparation

Organs were suspended in RPMI medium (Invitrogen A/S, San Diego, USA). Serum was collected after centrifugation at 10,000g for 10 min and stored at −20 °C until antibody titer analysis. The spleens were forced through a 100 μm nylon cell strainer (BD Falcon) and washed, first with PBS and subsequently with RPMI. The cells were resuspended in 2 ml complete RPMI (cRPMI) medium supplemented with 10% (v/v) fetal calf serum Superior (FCS, VWR-Bie & Berntsen). cRPMI consisted of RPMI and 10 mM HEPES buffer (Gibco™, Life Technologies) supplemented with 5 × 10^−6^ M β-mercaptoethanol (Sigma-Aldrich), 1% (v/v) penicillin/streptomycin (Invitrogen), 1% 100 mM sodium pyruvate (Invitrogen), 1 mM L-glutamine and non-essential amino acids (Invitrogen). The cells were counted using a NucleoCounter NC-100 (ChemoMetec, Lillerød, Denmark).

### 2.13 Meso Scale Discovery (MSD) assay

Spleen cells (10^6^ cells/well) were stimulated with 5 µg/ml unmodified LYS in sterile-Nunc U-bottom Nunclon Delta 96-well surface plates (Thermo Fisher Scientific), and the supernatants were harvested after 72 h incubation at 37 °C/ 5% CO_2_. The concentrations of secreted cytokines in the supernatants were measured using the MSD® U-Plex® Biomarker Group 1 (mouse) Multiplex Assays (Meso Scale Diagnostics, Rockville, MD, USA). A plate used to measure IFN-γ, IL-5, IL-10 and IL-17 was prepared by mixing each linker with its corresponding biotinylated antibody for 1 h at room temperature (RT) with *stop solution* (MSD® U-Plex®) added during the last 30 min of the incubation period. The plate was coated with the linker-antibody solution for 1 h with shaking at RT. A volume of 25 µl of the stimulated spleen cells was added to each well containing 25 µl *Diluent 41,* and the plate was incubated for 1 h at RT. Calibrators 5 and 7 was included on the plate as standards. After washing the plate with PBS supplemented with 0.05% (v/v) Tween 20, *detection antibody solution* diluted in *Diluent 45* was added to each well, and the plate was incubated for 1 h with shaking. *Read buffer T* was added to the plate before measuring the electrochemiluminescence using a Meso Sector Imager 2400 plate reader equipped with Discovery Workbench 4.0. MSD software (Meso Scale Diagnostics).

### 2.14 Antibody detection

ELISA was performed to measure the titers of Ag-specific IgG1 and IgG2a antibodies in serum. Nunc 96-well MaxiSorp plates (Thermo Fischer Scientific) were coated with 100 µl 1 µg/ml unmodified LYS in carbonate buffer (pH 9.6) (SSI Diagnostica, Hillerød, Denmark). The plates were incubated at 4 °C overnight and washed with 0.2% Tween (Merck) in PBS (pH 7.2, Gibco Life Technologies™). The plates were blocked for 1.5 h with 2% BSA in PBS. To give a 10-fold dilution curve, 1% BSA in PBS was added to each well and the serum was added. The plates were incubated at RT for 2 h. After washing the plates, they were incubated for 1 h with either horseradish peroxidase (HRP)-conjugated goat anti-mouse IgG1 (Invitrogen, Carlsbad, CA, USA) diluted 1:20,000 in 1% BSA in PBS or HRP-conjugated rabbit anti-mouse IgG2a (Invitrogen) diluted 1:5,000 in 1% BSA in PBS. The plates were developed with 100 μl/well 3,3′,5,5′-tetramethylbenzidine substrate (Kem-En-Tec Diagnostics, Taastrup, Denmark) for 10 min. The reaction was stopped by adding 0.2 M sulfuric acid (VWR, Radnor, PA, USA), and the plates were analyzed using a TECAN Sunrise™ ELISA reader (Tecan Trading) at OD_450_ nm corrected at OD_620_.

### 2.15 Statistical analysis

Statistical analysis was performed using the GraphPad Prism software v.8.2.1 (GraphPad, La Jolla, CA, USA). Cytokine responses and antibody responses were compared using one-way ANOVA with Dunnett’s multiple comparison test with unmodified LYS adjuvanted with CAF®01 as reference. A *p*-value <0.05 was considered statistically significant.

## 3 Results

### 3.1 Succinylation reduces the isoelectric point of lysozyme

**Fig. 1:**
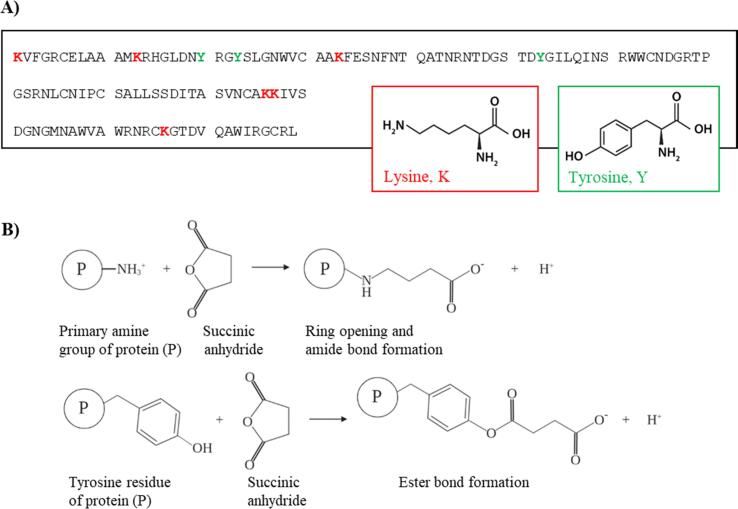
Succinylation of lysozyme. (A) The amino acid sequence of lysozyme with possible succinylation *i.e.* lysine residues (K) highlighted in red and tyrosine residues (Y) highlighted in green (structure sourced from NCBI). (B) Succinylation of a primary amino group and a tyrosine residue on a protein (P) with succinic anhydride at pH 8.0, resulting in the formation of an amide bond and an ester bond, respectively*.* (For interpretation of the references to colour in this figure legend, the reader is referred to the web version of this article.)

Depending on the initial pI of the investigated Ag, different methods can be applied. Succinylation and acetylation are methods to decrease the pI, whereas aminoamidation is a method used for increasing the pI of proteins [21]. We compared the three strategies on different Ags (data not shown) and succinylation was chosen as the preferred method to modify LYS for the present study. LYS, displaying a net positive charge at physiological pH, has been shown not to adsorb to CAF®01 at pH 7.4 [25]. Hence, to increase the attractive electrostatic interactions between LYS and CAF®01, LYS was chemically modified by succinylation to reduce the pI value [26]. Succinic anhydride reacts primarily with lysine and tyrosine residues. Therefore LYS contains a total of 10 reactive sites, including six lysine residues, three tyrosine residues and the N-terminal amine group (Fig. 1) [27], [28]. In previous studies a 100-fold molar excess of succinic anhydride relative to LYS was used to achieve complete succinylation of LYS [22]. In this study, the molar ratio of succinic anhydride to LYS used for reaction was titrated to investigate if partial succinylation, and hence partial reduction of the pI, was possible. Therefore, four different reactions with 100-fold, 10-fold, 5-fold and 0.5-fold molar excess of succinic anhydride relative to LYS, respectively, were carried out, resulting in the modified LYS analogues LYS-100, LYS-10, LYS-5 and LYS-0.5. In theory, both LYS-100 and LYS-10 will be fully succinylated at all 10 reactive sites, and the LYS-0.5 analogue will in average only contain one succinylated site for every two LYS molecules.

**Fig. 2:**
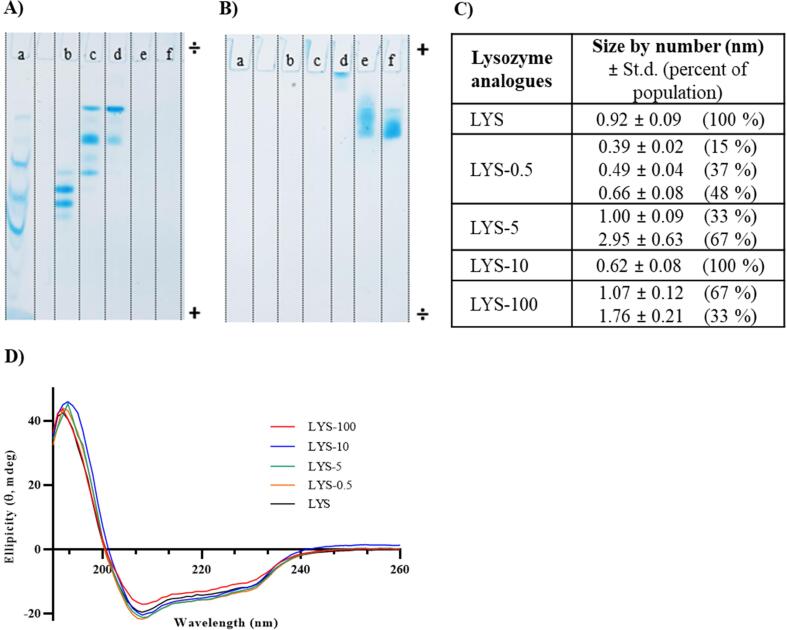
Physicochemical characterization of succinylated LYS analogue mixtures evaluated by (A) native gel electrophoresis and (B) native gel electrophoresis with reversed current direction. Both native gels were run on a 4–15% Mini-PROTEAN® TGX™ Precast Gel with premixed electrophoresis buffer containing 25 mM Tris, 192 mM glycine (pH 8.3) at 300 V for 20 min with 2 µg protein loaded onto each well: (a) the reference Precision Plus ProteinTM Standards, (b) LYS-100, (c) LYS-10, (d) LYS-5, (e) LYS-0.5 and (f) unmodified lysozyme (LYS). (C) The average number-weighted hydrodynamic diameter measured by dynamic light scattering (DLS) with standard deviation (st.d) and the percent of the particle population at each size. (D) The secondary structure of succinylated LYS analogue mixtures determined by far-UV circular dichroism. All generated spectra are an average of three scans. The shown spectra have not been smoothed.

First, the resulting succinylated LYS analogues were assessed by native PAGE at pH 8.3 (Fig. 2A). All succinylated LYS analogues, except LYS-0.5, displayed discrete and well-defined bands on the native gel, as the negatively charged analogues migrate towards the anode. This indicates that succinylation of LYS resulted in several LYS analogue mixtures with reduced pI and different degrees of succinylation. In addition, there was a correlation between the distance migrated into the gel and the molar excess of succinic anhydride applied for succinylation reaction (Fig. 2A). Unmodified LYS and LYS-0.5 were not visible on the gel, implying that the analogues in the LYS-0.5 mixture are positively charged and therefore they do not migrate towards the cathode, as expected for unmodified LYS. Therefore, the current direction was reversed, which resulted in migration of both unmodified LYS and modified LYS-0.5 into the gel towards the anode (Fig. 2B). This indicates that both unmodified LYS and the analogues in the LYS-0.5 mixture are positively charged at pH 8.3. A slight migration into the gel was also noted for LYS-5 upon reversing the current direction, indicating that this LYS analogue mixture contains one or several succinylated LYS analogues with pI values above the running buffer pH of 8.3.

### 3.2 Succinylation does not affect the secondary or tertiary structure of LYS

Chemical modification might alter the structural characteristics of proteins [26], which in turn can affect the immunogenicity of the Ags, especially in regard to humoral immunity that often involve conformational antibody epitopes. This is because highly specific B cell receptors interact directly with unprocessed Ags [29], whereas T cells recognize linear epitopes from processed Ags presented by APCs [30].

LYS is a structurally stable protein, and has previously been reported to undergo very little structural alterations upon chemical modification [28]. Nevertheless, the secondary and tertiary structure of the succinylated LYS analogue mixtures were investigated using DLS (Fig. 2C) and CD, respectively (Fig. 2D).

The hydrodynamic diameter of the modified LYS analogues showed no larger changes in particle sizes compared to unmodified LYS, although the size range for the modified LYS analogues is close to the detection limit of the Zetasizer NanoZS (0.3 nm). Some multimerization was expected for the partly-succinylated LYS analogues near neutral charge (zwitterionic) at pH 8.0, which might be the case for LYS-5 with an average hydrodynamic particle diameter of 2.95 nm (Fig. 2C). Since all modified LYS analogues were able to run on the SDS gel, this increase in size is assumed to be caused by reversible electrostatic interactions rather than a covalent binding.

Potential modifications in the secondary structure as a result of succinylation were investigated using far-UV CD. All modified LYS analogues displayed comparable spectra with a positive band at approximately 192–193 nm and a negative band around 208 nm, corresponding to a mixture of α-helix and β-sheet (Fig. 2D), as reported previously for unmodified LYS [28]. The differences in size distribution and secondary structure were negligible, hence, succinylation of LYS does not cause any major changes on the secondary structure of the protein.

### 3.3 The degree of succinylation can be controlled by varying the molar ratio of succinic anhydride to lysozyme used in the succinylation reaction

**Fig. 3:**
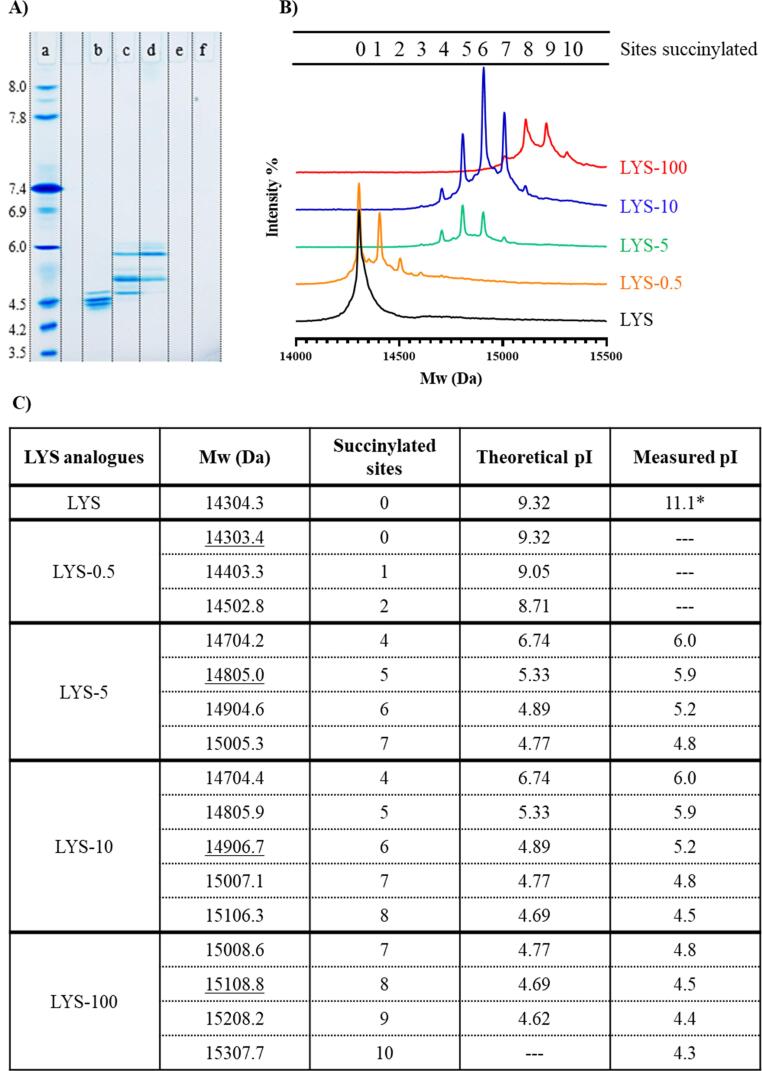
Degree of succinylation of LYS analogues investigated by (A) isoelectric focusing (IEF) with (a) the reference IEF Marker 3–10, SERVA Liquid Mix, reference values are included, (b) LYS-100, (c) LYS-10, (d) LYS-5, (e) LYS-0.5 and (f) unmodified lysozyme (LYS). (B) MALDI-TOF spectra with data provided by Alphalyse (Odense, Denmark), molecular weight (Mw) [M] + showed in Dalton (Da) as a function of the intensity (%) and (C) table summing the mass of the LYS analogues from MALDI-TOF in Da with the most intense peak underlined together with the corresponding sites of succinylation with the Mw of one succinyl group is 100.07 Da. The theoretical isoelectric point (pI) is included in the table. The pI is calculated using ExPasy Bioinformatics Resource Portal (http://web.expasy.org/protparam/) and replacing lysine and tyrosine residues with glutamate residues. The primary amine group has not been replaced. The actual pI of succinylated lysozyme is included and read of the IEF gel. *The practical pI of unmodified LYS is found in literature [20].

To assess the pI of the LYS analogue mixtures, an IEF gel was run (Fig. 3A). Considering the observed pI of LYS-100, LYS-10 and LYS-5 on the IEF gel, it is evident that the resulting pI decreases with increasing molar ratio of succinic anhydride to LYS. LYS-100 thus displayed a pI ≤ 4.8 with four distinct bands on both the native gel and the IEF gel, indicating that LYS has been succinylated to different degrees, resulting in a mixture of four different LYS analogues with varying pI values of 4.3, 4.4, 4.5 and 4.8, respectively (Fig. 3A). Similarly, both LYS-10 and LYS-5 displayed several bands on the IEF gel. For both mixtures, strong bands were apparent at pI values of 5.2 and 5.9. LYS-10 displayed an additional band at a pI value of 4.8, similar to a band in LYS-100, probably representing a LYS analogue with similar degree of succinylation. However, the band at a pI of 4.8 value was very weak for LYS-5. Unmodified LYS did not migrate into the IEF gel (pI span 3.5–8) (Fig. 3A) and LYS-0.5 was not visible on the IEF gel either, which suggests pI values above 8.0 and therefore outside the IEF detection range.

The theoretical pI value of the succinylated LYS analogues were calculated using the ProtParam online tool by replacing lysine and tyrosine residues in LYS with glutamate residues, which contains a carboxylic acid comparable to the one introduced by succinylation (Fig. 1B, Suppl. Fig. 1). Replacing all six lysine residues and the three tyrosine residues resulted in a theoretical pI value of 4.62. It was not possible to replace the primary amine at the N-terminal of the protein with a carboxylic acid group in the theoretical calculation of the pI. Despite this, the calculated isoelectric points were close to those observed on the IEF gel (Fig. 3A, C), suggesting that each band on the IEF gel represent LYS analogues displaying different degrees of succinylation. The order in which the amino acids are replaced with glutamate residues does not affect the calculated pI values.

The degree of succinylation was confirmed by measuring the molecular weight of the succinylated LYS analogue mixtures using MALDI-TOF peptide mass fingerprinting (Fig. 3B). The mass of unmodified LYS was determined to 14,304.3 Da. LYS-100 displayed four peaks with masses of 15,008.6, 15,108.8, 15,208.2 and 15,307.7 Da, respectively. With a succinyl group mass of 100.07 Da, the four different LYS analogues in the LYS-100 mixture are succinylated in seven, eight, nine and ten sites, respectively. This is consistent with the four bands identified by IEF and suggests that seven amine groups are succinylated for the LYS analogue with a pI ~ 4.8 band. Additional succinylation of the phenol groups present in the three tyrosine residues contribute to reducing the pI value for each additional band to 4.5, 4.4 and 4.3, respectively (Fig. 3C).

Modified LYS-10 displayed five peaks in the MALDI-TOF spectrum (Fig. 3B) with the three most dominant peaks corresponding to succinylation at five, six and seven sites, respectively, which were evident as three distinct bands on the IEF gel. The additional two and less intense peaks correspond to LYS succinylated at four and eight sites, respectively. Both LYS-100 and LYS-10 displayed abundant analogues succinylated at seven sites, which was predicted from the band in the IEF gel at pI 4.8 that they both shared. Even though it was theoretically possible, LYS-10 was not fully succinylated at all 10 sites.

The modified LYS-5 contained analogues, which were succinylated primarily in five and six sites, with less abundant analogues succinylated in four and seven sites. On the IEF gel there were two distinctive bands corresponding to the bands representing LYS analogues succinylated in five and six sites, respectively. There was an additional weak band at a pI value of approximately 6.0 on the IEF gel, which might correspond to a LYS analogue succinylated in four sites. This correlates approximately with a theoretical pI value of 6.74 (Fig. 3C).

The LYS-0.5 analogue mixture displayed three peaks and the most intense peaks corresponded to unmodified LYS. However, there were fractions of LYS analogues succinylated at one or two sites. The theoretical pI values of the analogues present in the LYS-0.5 mixture were 9.05 and 8.71, respectively, and thus outside of the IEF gel reference area (pI 3.5–8) and the experimental pI values of the analogues in the LYS-0.5 mixture could not be determined.

### 3.4 Succinylation of lysozyme results in increased adsorption to CAF®01

**Fig. 4:**
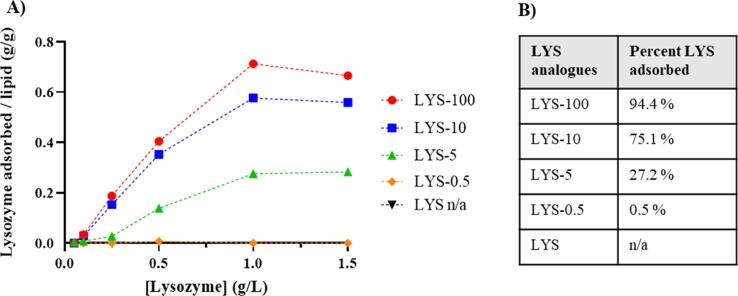
Adsorption of lysozyme to CAF®01. (A) Adsorption ratio of lysozyme to CAF®01 shown as the fraction of succinylated LYS analogue adsorbed per gram lipid as a function of the added antigen concentration (g/L). Data represent mean values of three technical replicates (n = 1). Values below zero were corrected to zero. (B) Percentage of LYS adsorbed to CAF®01 in each LYS analogue containing different concentrations (0.25–1 g/L) of LYS. Data represent mean values of three technical replicates due to shortage of succinylated LYS.

The modified LYS analogue mixtures were mixed with CAF®01 at a ratio of 0–1.5 g/L LYS to CAF®01 to quantify the adsorption onto the cationic adjuvant, which was measured as the fraction of unbound LYS in the supernatant after ultracentrifugation. The adsorption to CAF®01 was inversely proportional to the pI value of the succinylated LYS analogues (Fig. 4A). CAF®01, mixed with unmodified LYS, deposited at the air–water interphase, and it was thus not possible to sample the supernatant, indicating that there is no attractive electrostatic interactions between unmodified LYS and CAF®01. The same has been reported previously using the same ultracentrifugation method [25].

The LYS-0.5 and LYS-5 analogue mixtures displayed the lowest degree of adsorption onto CAF®01 of 0.5% and 27.2% of added protein adsorbed, respectively (Fig. 4B). Additionally, the degree of adsorption of LYS-100 was higher than the adsorption of LYS-10 with 94.4 and 75.1% of added protein, respectively, adsorbed onto CAF®01 and it was evident that the adsorption of succinylated LYS increased with decreasing pI (Fig. 4A, B). The supernatants from the adsorption study were subjected to IEF, which indirectly confirmed that all analogues in the LYS-100 analogue mixture were adsorbed to CAF®01, since no bands appeared on the gel. In comparison, the supernatants from the LYS-10 and LYS-5 analogue mixtures still contained LYS analogues succinylated at five sites (pI ≈ 6) or less (Suppl. Fig. 2). This indicates that the modified LYS analogues with pI values ≤ 6 are fully adsorbed to CAF®01.

### 3.5 Reducing the pI of LYS enhances CAF®01-induced LYS-specific Th1 and Th17 cell responses

**Fig. 5:**
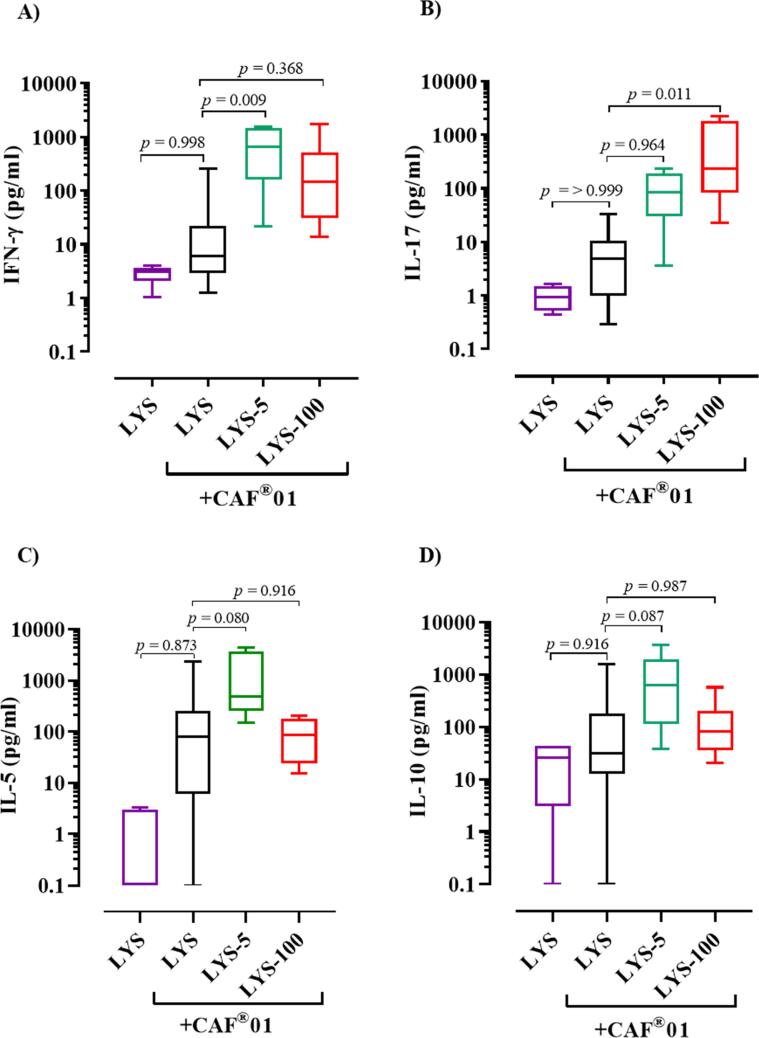
Cell-mediated immune response. (A–D) Electrochemoluminiscence assay by MSD of supernatants from splenocytes from female BALB/C mice harvested three weeks post s.c. immunization with three times 200 µl 5 µg Ag/dose vaccine and restimulated with unmodified LYS for three days. (A) IFN-γ, (B) IL-17, (C) IL-5 and (D) IL-10 cytokine secretion. Data are shown as mean values ± max/min. Data are representative of three independent experiments. Statistical analysis was performed using one-way ANOVA with Dunnett’s multiple comparison test against the control group (LYS + CAF®01). P-values are stated in the figures.

In order to assess the effect of Ag charge on the immune responses, mice were vaccinated with CAF®01-adjuvanted unmodified LYS (non-adsorbed), the intermediately adsorbed LYS-5 analogue mixture and the strongly adsorbed LYS-100. Unadjuvanted, unmodified LYS was used as a negative control. The mice were vaccinated three times with two weeks interval and the study was terminated three weeks after the last immunization. Single-cell suspensions of splenocytes were restimulated with unmodified LYS and the secretion of IFN-γ, IL-5, IL-17 and IL-10 in the supernatant was measured as general markers for Th1, Th2, Th17 and T_REG_ induction, respectively, with an electrochemoluminiscence assay (MSD assay) (Fig. 5A–D).

The IFN-γ responses for the group immunized with LYS-5 adjuvanted with CAF®01 were significantly increased compared to the responses for the group immunized with unmodified LYS adjuvanted with CAF®01 (p = 0.009). There was also a tendency of increased IFN-γ responses in the group dosed with LYS-100 adjuvanted with CAF®01 compared to unmodified LYS group adjuvanted with CAF®01, although the difference was not statistically significant (p = 0.368) (Fig. 5A). In addition, the IL-17 response, which is a hallmark of CAF®01-induced immune responses [31], was significantly higher in the group immunized with adjuvanted LYS-100 (p = 0.011) as compared to unmodified LYS adjuvanted with CAF®01 (Fig. 5B). Notably, unmodified LYS induced comparable low IFN-γ and IL-17 responses when administered alone and when formulated with CAF®01. The results thus show that increased adsorption between LYS and CAF®01 resulted in increased Th1 and Th17 responses. When comparing splenocytes that were restimulated with unmodified LYS with splenocytes restimulated with modified LYS-5, we found that splenocytes from LYS-100 + CAF®01 vaccinated mice responded stronger to unmodified LYS compared to modified LYS-5 with respect to IFN-γ, whereas the IL-17 responses were comparable (Suppl. Fig. 3). Whether this small but consistent difference is assay-related or due to changes in T cell recognition of the antigen warrants further investigation.

Reducing the pI of LYS also affected the Th2 and T_REG_ related responses, and there was a tendency of increased IL-5 and IL-10 levels for the group vaccinated with the adjuvanted LYS-5 analogue compared to adjuvanted, unmodified LYS (not statistically significant, p = 0.080 and p = 0.087, respectively), whereas adjuvanted LYS-100 induced Th2 responses similar to adjuvanted LYS (Fig. 5C, D). This suggest that Th2 and T_REG_ responses benefits from partial adsorption but not from strong adsorption of the Ag to CAF®01, although this has to be investigated further.

### 3.6 Strong adsorption of lysozyme to CAF®01 does not increase antibody responses *in vivo*

**Fig. 6:**
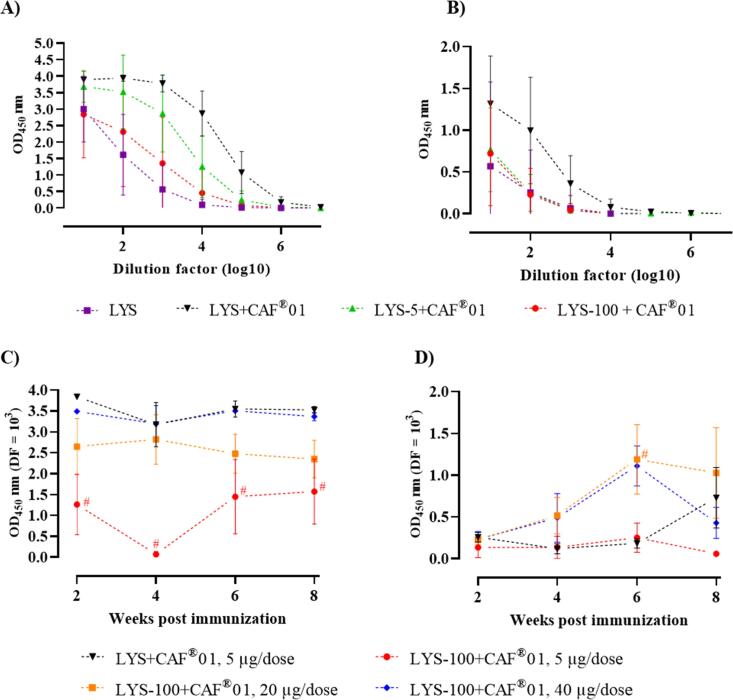
Antibody responses. Unmodified LYS-specific (A) IgG1 and (B) IgG2 antibodies were detected 3 weeks post immunization using an antibody enzyme-linked immunosorbent assay (ELISA). Female BALB/C mice were injected s.c. with 200 µl 5 µg Ag/dose of unmodified LYS alone (n = 5) or with CAF®01 or LYS-5 + CAF®01 or LYS-100 + CAF®01 (n = 8) three times, with 14 days between immunizations. Blood was harvested on day 49 (3 weeks p.i.) and the serum analyzed. Data are shown as mean ± SD. (C) and (D) Detection of unmodified LYS-specific (C) IgG1 and (D) IgG2a antibodies 2, 4, 6 and 8 weeks after the last immunization by ELISA. Data shown represent mean values ± SEM at a dilution factor of 10^3^. Female BALB/C mice were injected s.c. with 5 µg/dose LYS + CAF®01, 5 µg/dose LYS-100 + CAF®01, 20 µg/dose LYS-100 + CAF®01, or 40 µg/dose LYS-100 + CAF®01 (n = 4) three times, with 14 days between immunizations. Blood was harvested, and the serum was analyzed. Statistical analysis was performed using one-way ANOVA with Dunnett’s multiple comparison test for each time-point with LYS + CAF®01 (5 µg/dose) as reference. Significant differences [P-value < α (0.05)] are marked with #. The optical density (OD) was measured with an absorbance of 450 corrected at 650 nm.

The LYS-specific IgG1 and IgG2a titers in mice immunized with unadjuvanted LYS and CAF®01-adjuvanted LYS, LYS-5 and LYS-100 were assessed by ELISA. When adjuvanted with CAF®01, unmodified LYS induced higher IgG1 and IgG2a titers than LYS-100, which indicates that increasing the adsorption of LYS to the adjuvant results in reduced antibody responses. LYS-5, which displays an intermediate degree of adsorption to CAF®01, induced intermediate antibody responses (Fig. 6A, B). Coating the ELISA plates with the LYS-100 analogue instead of unmodified LYS gave comparable results (Suppl. Fig. 3). Hence, the induction of Ag-specific antibodies apparently correlate with the presence of unbound Ag, suggesting that the availability of non-adsorbed Ag for presentation to B cells is a prerequisite for their activation and antibody induction. Another possibility could be that the succinylation changes the B cell epitopes present in LYS, eventually resulting in a less immunogenic protein. To investigate this further, we compared unadjuvanted, unmodified LYS with unadjuvanted LYS-100 and found that there was a slightly reduced recognition of LYS-100 from Abs generated by vaccination with unmodified LYS, whereas those Abs generated by vaccination with LYS-100 recognized both LYS-100 and unmodified LYS equally well (Suppl. Fig. 7). Even though the Ab levels induced by the Ags alone were too low to conclude from, this suggest that Ag succinylation has the potential to change the Ab induction, potentially through modification of the epitopes. In a follow-up experiment, the dose of LYS-100 adjuvanted with CAF®01 was increased to 20 and 40 µg, respectively. According to the adsorption experiment, the Ag is fully adsorbed to CAF®01 at these doses as they are within the linear part of the curve (Fig. 4A). The development of antibody responses was monitored over a period of eight weeks after the last immunization to follow the kinetics of the antibody responses. Increasing the Ag dose of LYS-100 to 40 µg resulted in IgG1 levels, which was comparable to the levels measured for the 5 µg dose of unmodified LYS adjuvanted with CAF®01 throughout the study (Fig. 6C). Increasing the LYS-100 dose to 20 or 40 µg also resulted in increased IgG2a responses, which were comparable to the levels for the 5 µg/dose of unmodified LYS (Fig. 6D, Suppl. Fig. 4, Suppl. Fig. 5). Thus, the succinylated LYS analogues are able to induce antibody responses at high Ag doses.

## 4 Discussion

The mechanism of action for the most widely used adjuvant system based on aluminum salts has been ascribed to its ability to adsorb Ags and form an Ag depot at the SOI, which results in sustained release of Ag and thus prolonging the Ag exposure to the immune system [4], [5]. On the other hand, transport of unprocessed Ag to the draining LNs is a prerequisite for B cell activation and differentiation to antibody-producing plasma cells [32]. Therefore, immunization with Ags, which are retained at the SOI, may cause a limited presentation to the B cells and consequently induce a lower or delayed antibody response. The influence of Ag adsorption to adjuvants, as well as the Ag-depot formation after administration of vaccines, has been studied thoroughly [8], [17], [25], [33], [34], [35]. For alum-based vaccines it has been demonstrated that strong binding of Ag to aluminum hydroxide reduces antibody responses [33], [36]. In contrast, in a recently published study serine residues were introduced in a bona fide vaccine Ag to increase the binding strength to aluminum hydroxide, eventually resulting in retention of the modified Ag at the SOI for up to 14 days. The increased retention increased Ag-specific IgG titers compared to the responses induced by formulations with unmodified/non-adsorbed Ag [37]. Similar conclusions were made in a study introducing phosphonate (C-PO_3_) groups to the LYS protein, which increased adsorption to aluminum hydroxide and resulted in increased antibody titers [38]. Thus, discrepancies exist in whether a strong adsorption of Ag to alum-based adjuvants is indeed needed for induction of antibody responses. Further adding to this complexity is that some studies have shown that Ags, which do not adsorb to aluminum-based adjuvants prior to immunization, can still be trapped within adjuvant aggregates at the SOI and thereby retain the Ag as a depot [9]. Overall, Ag adsorption to aluminum-based adjuvants and the effects on the induced immune responses appears to be a balance that depends on the nature and complexity of the Ag, including its ability to form electrostatic, hydrophobic and ligand-exchange interactions with the adjuvant [39].

In contrast to studies investigating how adsorption to adjuvants influence humoral immune responses, only few studies have addressed how Ag adsorption to adjuvants influences T cell responses.

While aluminum-based adjuvants are generally poor inducers of cell-mediated immune responses, cationic liposomes containing the Mincle agonist TDB (CAF®01) induce strong T cell responses [40]. The role of Ag adsorption to the CAF®01 adjuvant in activation of the immune system has previously been studied by varying the liposome composition [15] and by comparing different Ags [20]. However, limitations of these strategies is that different lipid components likely have varying immune activating mechanisms not related to the Ag association. To circumvent these limitations, we modified the net charge of a single Ag to varying degrees. Hereby, the degree of adsorption to CAF®01 can be tightly controlled to study the importance of Ag/CAF®01 adjuvant adsorption on the resulting immune response.

Our studies demonstrate that the pI has an effect on the adsorption of the Ag to the CAF®01 adjuvant with an inverse correlation between pI and level of association. In contrast to unmodified LYS (pI ~ 11), the LYS-100 analogue (pI ~ 4.3–4.8) was almost fully adsorbed to CAF®01, similarly to what was previously reported for proteins in the same pI range, *e.g.* BSA (pI = 4.6), ovalbumin (pI = 4.5) and Ag85b-ESAT-6 (pI = 4.6) [17], [25]. Importantly, the effect of the different Ag adsorption levels prior to immunization was directly reflected in the Th17 and Th1 responses, which benefitted from a strong adsorption between Ag and adjuvant. This is in line with published studies showing that a high degree of adsorption prior to immunization is important for the induction of CAF®01-induced Th1/Th17 immune responses [11]. Other studies have shown that depot formation is important for CAF®01 adjuvanted vaccines to elicit Th1 and Th17 responses [11], [15], [16]. Using I^125^ labelling of the net negatively charged tuberculosis Ag H1, with more than 80% of H1 adsorbed to CAF®01 prior to immunization, 21% was still retained at the SOI 14 days post immunization [15]. Even though we did not determine the degree of retention of LYS-100 at the SOI in this study, the comparable properties of H1 and LYS-100 (H1: pI 4.6, >80% adsorption vs. LYS-100: pI 4.4–4.8, 94% adsorption) suggest that they may have comparable kinetics. Retention of Ag with CAF®01 at SOI prevented the early targeting of lymph node DCs by non-adsorbed Ag that do not facilitate simultaneous activation of the DCs and results in a temporary anergy, which again inhibits T cell induction [11]. This could be an explanation for why unmodified LYS, which is weakly adsorbed to CAF®01, elicit lower T cell responses than LYS-5 and LYS-100, where lower amounts of free Ag is expected to reach the draining LNs. The present study thus further supports the hypothesis that adsorption of Ag to CAF®01 is important for Th1/17-cell induction. Interestingly Th2 responses were most strongly induced by LYS-5 + CAF®01 vaccination, where the Ag binding is only partial. That Th2 responses decreases with stronger binding might be due to the intrinsic inhibitory effect of Th1/17 cytokines on Th2 induction. However this potential relation needs further investigation.

One limitation with the succinylation could be that the modification can affect the affinity of specific T cell epitopes with MHC and thus influence the T cell responses. In LYS, particularly the residues 107–116, which have been identified as the immunodominant T cell epitope [41], contains lysine and could thus be modified by succinylation, which may again change T cell receptor binding affinity. Previous reports have not investigated modifications of this kind in lysine residue 116 but have reported that substitutions at positions 113 (Asn-Lys), 114 (Arg-His), or 115 (Cys-Ala) abrogate the ability of LYS-(105–120) to activate T cells. Substitutions at residues 113 and 115 affect T-cell recognition but not the binding to MHC, whereas Arg-His substitution at position 114 impairs the capacity of the peptide to interact with the MHC molecules. A concern could thus be that modified LYS-induced T cells did not recognize the unmodified antigen, but when comparing unmodified LYS restimulation of lymphocytes from vaccinated animals with modified LYS-5 restimulation (Suppl. Fig. 3), we found that LYS-5 induced lower IFN-γ and IL-17 responses than LYS, even though the mice had been vaccinated with LYS-100. This could indicate that the epitopes from LYS-5 *in vitro* had a lower T cell receptor (TCR) affinity than unmodified LYS. However, since the vaccination-induced T cells induced by LYS-100, recognized unmodified LYS during restimulation, this suggest that the *in vivo* processing of the Ag eliminates this difference in TCR affinity observed *in vitro*, potentially by proteases cleaving the amide bond by hydrolysis to restore the primary amine. Thus, overall the succinylation does not change the recognition of the unmodified epitope.

Although our results suggest that strong adsorption of Ag to the adjuvant results in increased Th1 and Th17 responses, the antibody responses appeared not to benefit from increased adsorption. Hence, CAF®01-adjuvanted unmodified LYS promoted a higher antibody response compared to the intermediately complexed LYS-5 and the strongly adsorbed LYS-100 analogue. Our results are in line with a previous publication demonstrating that a very tight complexation between Ag and adjuvant is unfavorable for antibody responses [33]. The immune responses generated by restimulation with unmodified LYS are generally higher than those of LYS-100 (Suppl. Fig. 7). This could be because LYS-100 has weaker B cell epitopes or because the cationic nature of unmodified LYS will make it stick to interstitial proteins like albumin, and thus potentially improve presentation to the B cells and APCs in the dLN, whereas modified LYS has a bigger risk of getting cleared from the system. However, antibody responses could be boosted by increasing the dose of Ag strongly adsorbed to CAF®01 (Fig. 6C, D). Even though the adsorption studies showed that LYS-100 was fully adsorbed to CAF®01, interstitial proteins at the SOI might compete for the binding to the cationic liposome and displace Ag more readily when more Ag is present [35], [42]. Thus, the higher dose of Ag results in more Ag being displaced after immunization, indicating that non-adsorbed Ag draining to the LNs is favorable for B cell activation.

Overall, this study illustrates that Ag-adjuvant interactions affect both cell-mediated and humoral immune responses and should be considered in the early stages of vaccine design. Whether it is solely the co-localization of the antigen and the adjuvant that mediate the increased Th1/17 responses or whether the depot formation at the site of injection also play a role cannot be deduced from the present study. Furthermore, succinylation may be used as a strategy for modifying vaccine Ags for improved cell-mediated immune responses.

## 5 Conclusion

This study demonstrates that increasing attractive electrostatic interactions between Ag and CAF®01 adjuvant by varying the charge of one single Ag and thus its complexation to the CAF®01 affects the immune responses induced by the vaccine in mice. LYS was succinylated to different degrees, resulting in LYS analogues displaying pI values down to 4.3. Adsorption studies showed a clear inverse correlation between the pI value and adsorption to CAF®01. The *in vivo* immunization studies confirmed that the degree of Ag adsorption to CAF®01 directly affects the immune response as a strong Ag adsorption to CAF®01 correlates directly with the induction of Th1 and Th17 responses, but inversely with antibody titers. Hence, this study emphasizes the importance of considering the physicochemical properties of both Ag and adjuvant when designing new vaccines as the Ag adsorption to adjuvant is likely to affect both humoral and cell-mediated immune responses.

## Funding

This publication is part of a TRANSVAC2 project that has received funding from the European Union’s Horizon 2020 research and innovation programme under grant agreement No 730964.

## Declaration of Competing Interest

The authors declare the following financial interests/personal relationships which may be considered as potential competing interests: ‘KW, STS, IR, GKP and DC are employed by Statens Serum Institut, a nonprofit government research facility, which holds patents on the cationic liposomal adjuvants (CAF). All other authors report no potential conflicts’.
