# Supplementary material for: Adsorption of protein antigen to the cationic liposome adjuvant CAF®01 is required for induction of Th1 and Th17 responses but not for antibody induction
Source: Eur J Pharm Biopharm. 2021 Aug;165:293–305. doi: 10.1016/j.ejpb.2021.05.020 (PMC8212872; doi:10.1016/j.ejpb.2021.05.020)
Supplement: Supplementary data 1 [file mmc1.docx]

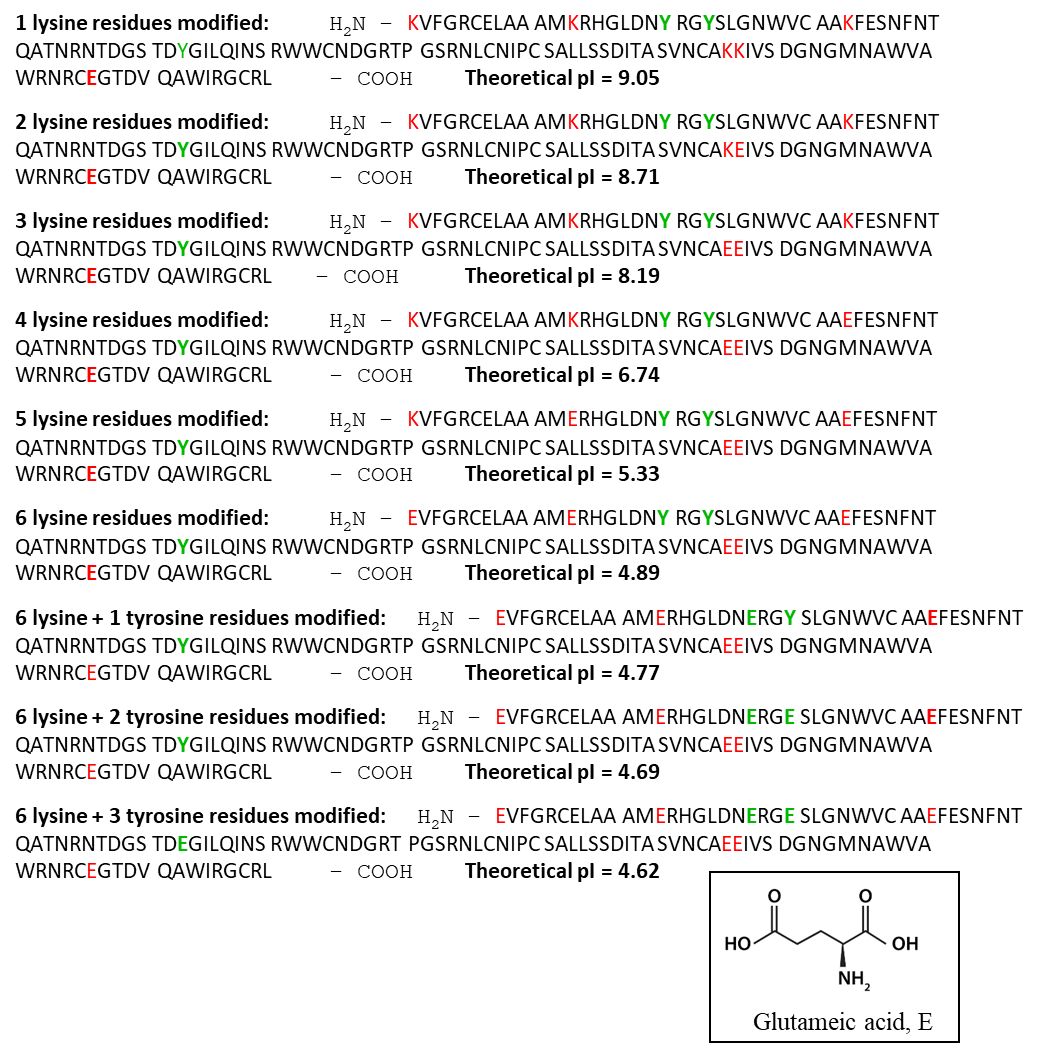


Suppl. figure 1. Amino acid sequence of modified lysozyme analogues with lysine (K, red) and tyrosine (Y, green) residues replaced with glutameic acid (E) residues and their theoretical isoelectric point (pI) calculated using ExPasy Bioinformatics Resource Portal [24]. The order in which K and Y are substituted with E does not affect the calculated pI values.


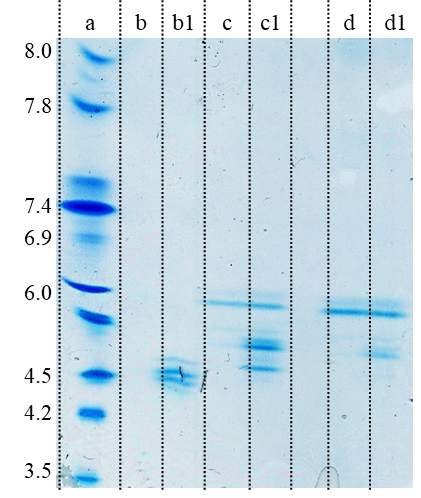


Suppl. figure 2. Determination of lysozyme components in the supernatants from the adsorption study showing lysozyme left in the supernatant and not complexed with CAF^®^01. Measured as antigen in the supernatant in an ultra-centrifuged formulation containing both liposome and antigen investigated on an isoelectric focusing (IEF) gel with a) the reference IEF Marker 3-10, SERVA Liquid Mix, reference values are included, b) supernatant LYS-100, b1) unadjuvanted LYS-100, c) supernatant LYS-10, c1) unadjuvanted LYS-10, d) supernatant LYS-5 and d1) unadjuvanted LYS-5.


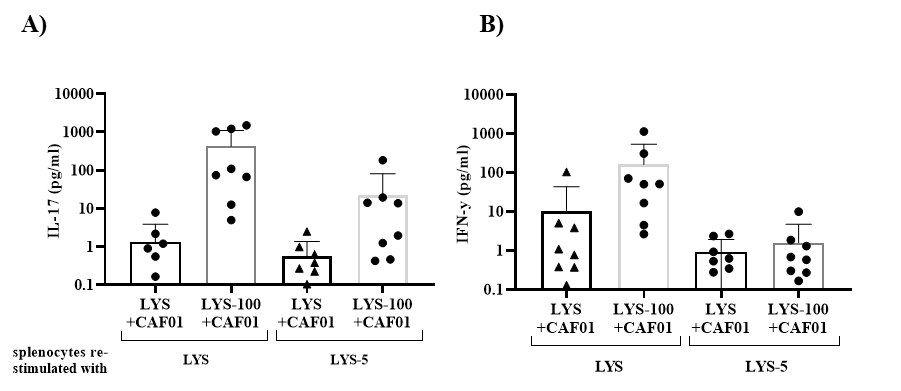


Suppl. figure 3. Cell-mediated immune response when restimulated with either LYS or LYS-5. A-B) Electrochemoluminiscence assay by MSD of supernatants from spleenocytes from female BALB/C mice harvested three weeks post s.c. immunization with three times 200 µl 5 µg Ag/dose vaccine and restimulated with LYS or LYS-5 for three days. A) IL-17 and B) IFN-γ cytokine secretion. Data are shown as mean values ± SD.


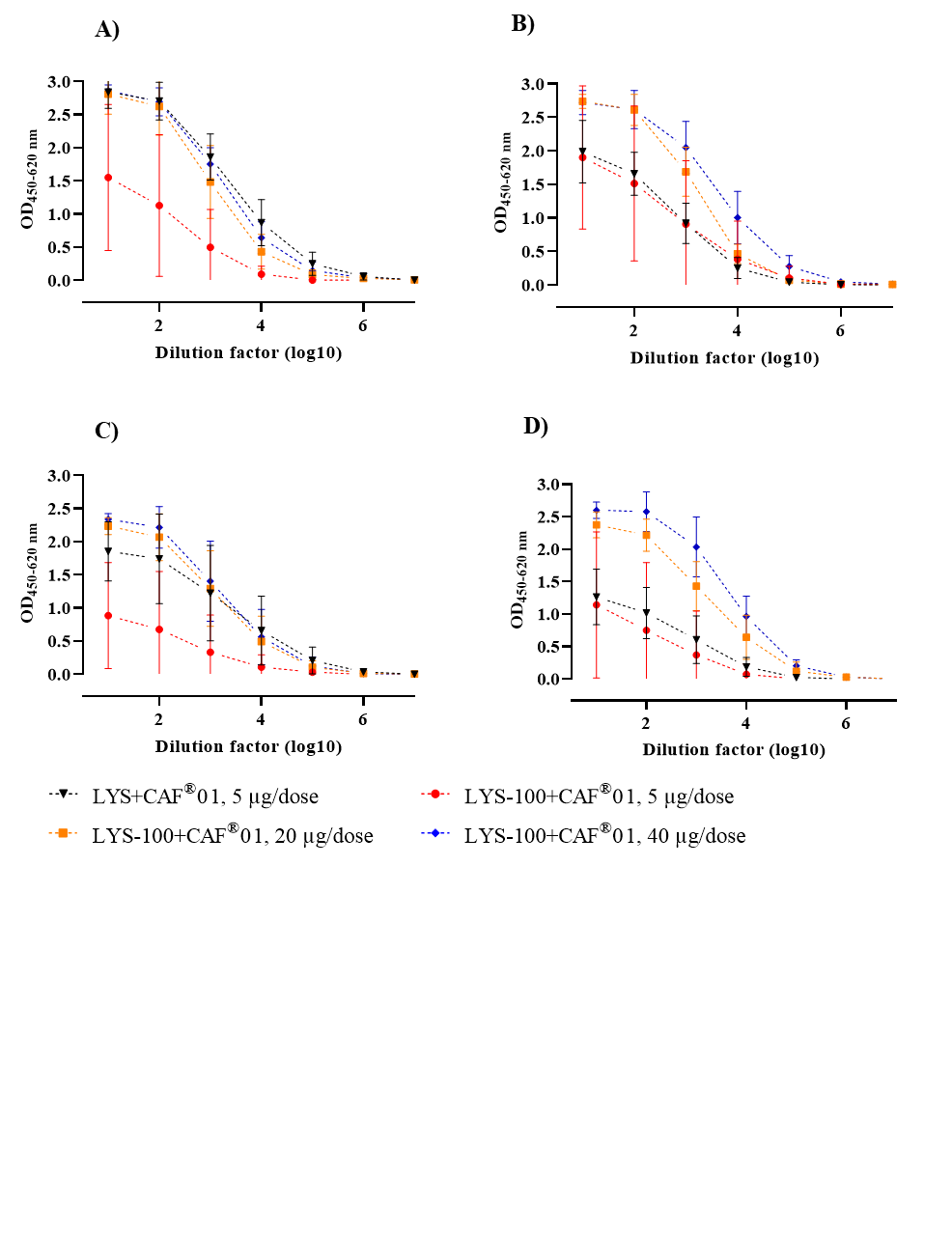


Suppl. figure 4. Comparison of antibody responses with the detection of unmodified lysozyme (LYS)- and LYS-100- specific IgG antibodies with antibody enzyme-linked immunosorbent assay (ELISA) assay, data shown as mean ± SD. Female mice were injected s.c. with 5 µg/dose LYS + CAF^®^01, 5 µg/dose LYS-100 + CAF^®^01, 20 µg/dose LYS-100 + CAF^®^01 or 40 µg/dose LYS-100 + CAF^®^01 (n = 4) three times, with 14 days between immunizations. Blood was harvested and the serum analyzed. The OD was measured with an absorbance of 450 nm corrected at 650 nm. A) serum harvested 2 weeks p.i. tested with unmodified LYS, B) serum harvested 2 weeks p.i. tested with LYS-100, C) serum harvested 4 weeks p.i. tested with unmodified LYS and D) serum harvested 4 weeks p.i. tested with LYS-100.


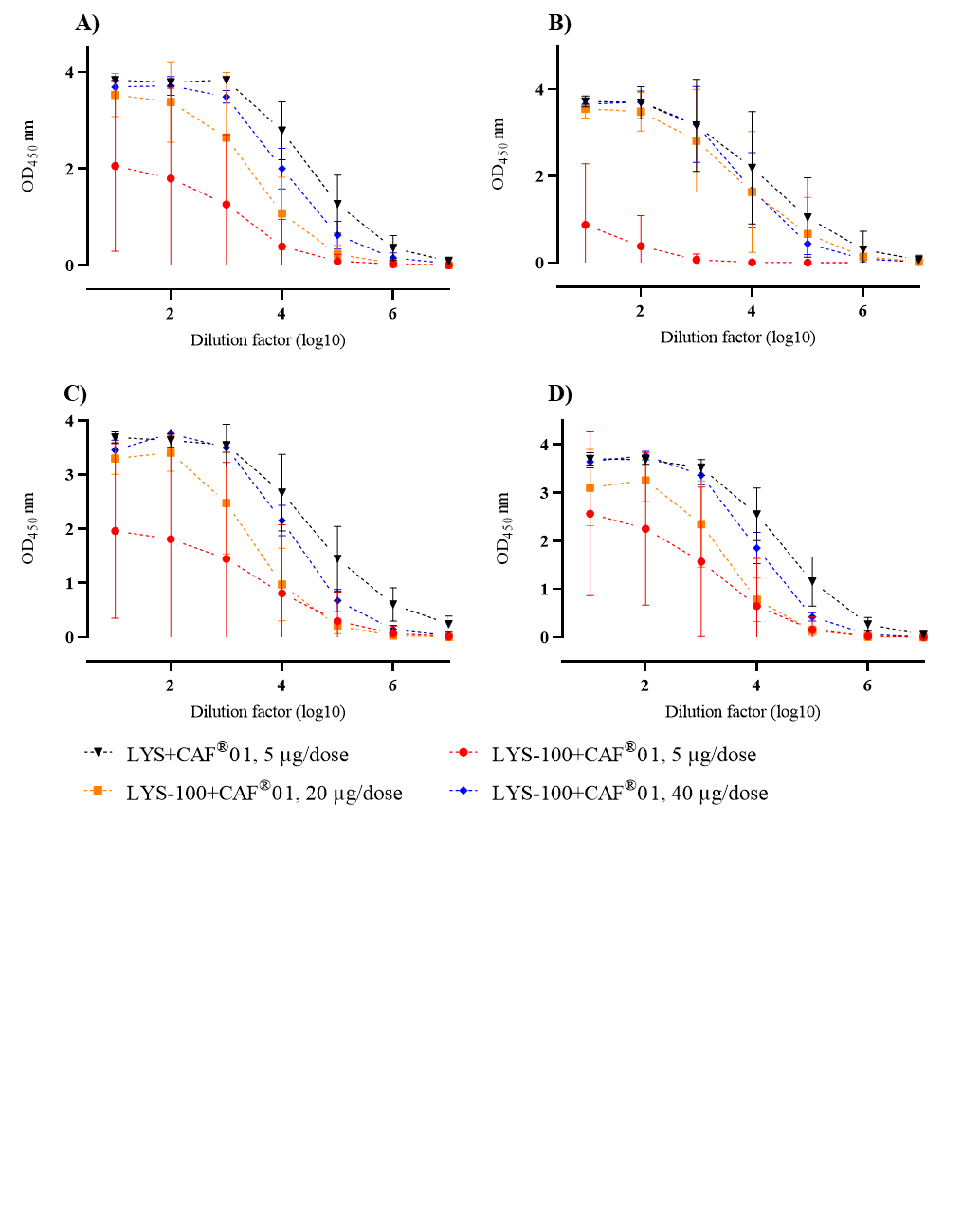


Suppl. figure 5. Titration curves of unmodified LYS-specific IgG1 antibodies with antibody Enzyme-linked Immunosorbent Assay (ELISA) assay, data shown as mean ± SD. Female mice were injected s.c. with 5 µg/dose LYS + CAF^®^01, 5 µg/dose LYS-100 + CAF^®^01, 20 µg/dose LYS-100 + CAF^®^01 or 40 µg/dose LYS-100 + CAF^®^01 (n = 4) three times, with 14 days between immunizations. Blood was harvested, and the serum was analyzed. The OD was measured with an absorbance of 450 nm corrected at 650 nm. The serum was harvested A) 2 weeks p.i., B) 4 weeks p.i. C) 6 weeks p.i. and D) 8 weeks p.i.


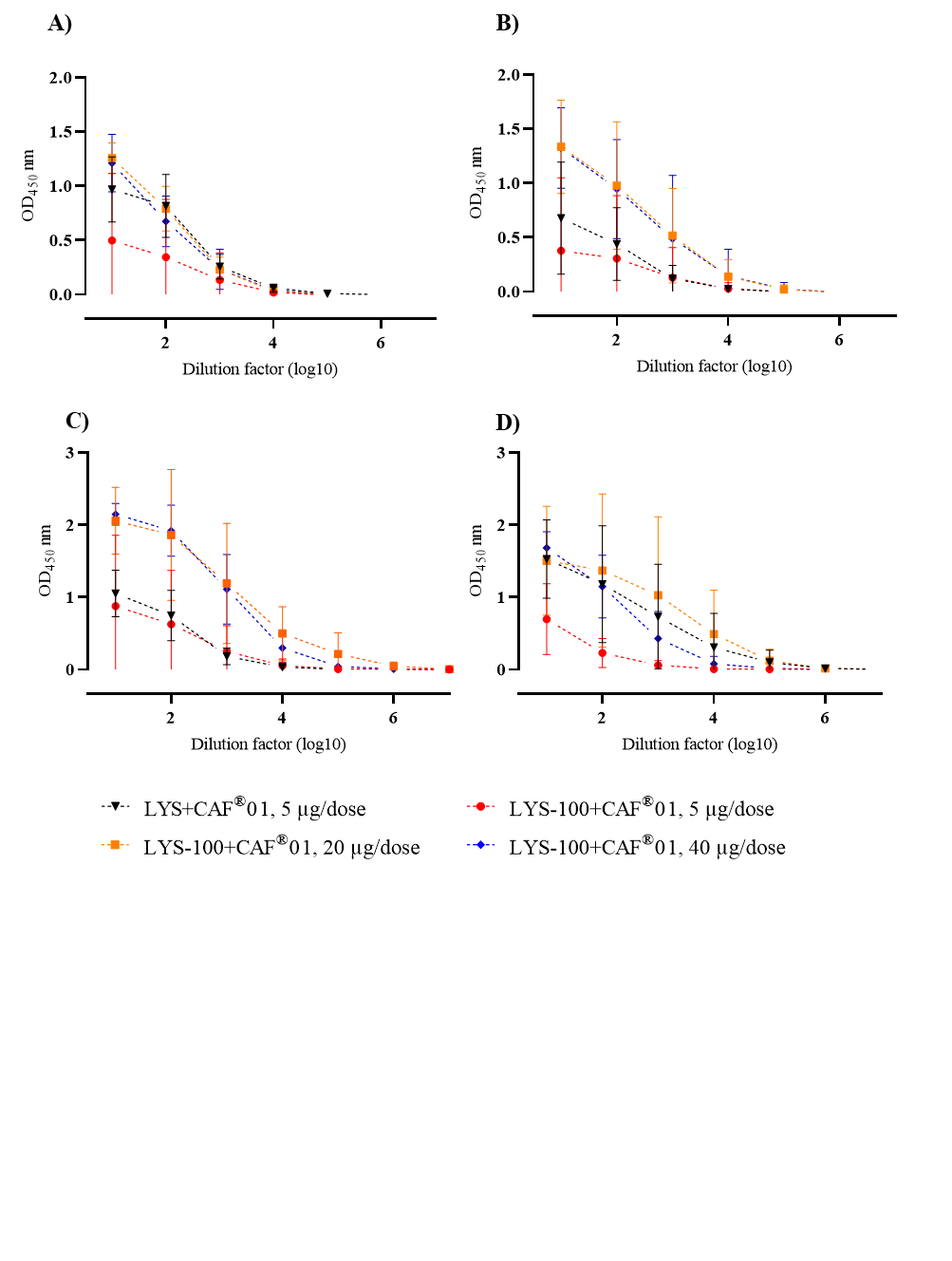


Suppl. figure 6. Titration curves of unmodified LYS-specific IgG2a antibodies with antibody Enzyme-linked Immunosorbent Assay (ELISA) assay, mean ± SD. Female mice were injected s.c. with 5 µg/dose LYS + CAF^®^01, 5 µg/dose LYS-100 + CAF^®^01, 20 µg/dose LYS-100 + CAF^®^01 or 40 µg/dose LYS-100 + CAF^®^01 (n = 4) three times, with 14 days between immunizations. Blood was harvested, and the serum was analyzed. The OD was measured with an absorbance of 450 nm corrected at 650 nm. The serum was harvested A) 2 weeks p.i., B) 4 weeks p.i. C) 6 weeks p.i. and D) 8 weeks p.i.


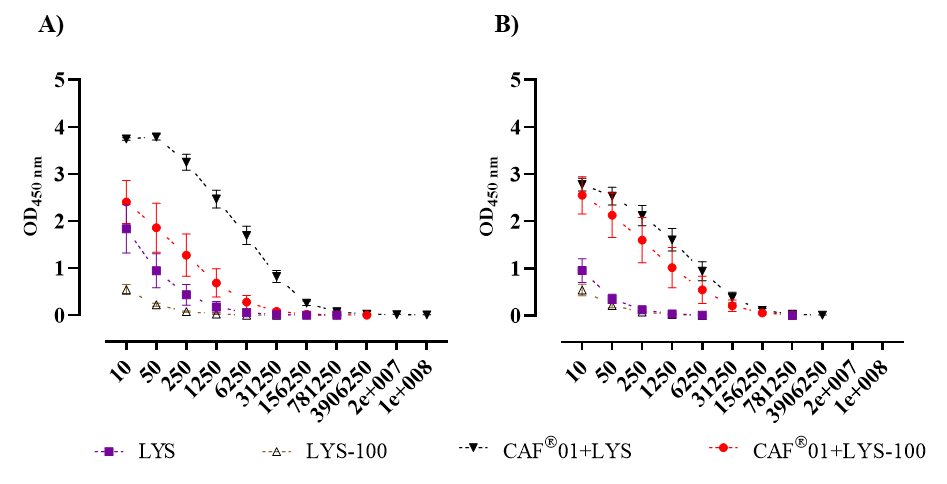


Suppl. figure 7. Titration curves of LYS- vs. LYS-100-specific total IgG antibodies measured with antibody Enzyme-linked Immunosorbent Assay (ELISA) assay, mean ± SD. Female mice were injected s.c. with 5 µg/dose unadjuvanted or adjuvanted LYS and LYS-100 or adjuvanted LYS + CAF®01 or LYS-100 + CAF®01 three times, with 14 days between immunizations. Blood was harvested three weeks post the last immunization, and the serum was analyzed. The OD was measured with an absorbance of 450 nm corrected at 650 nm. ELISA plates were coated with A) unmodified LYS-specific IgG antibodies and B) LYS-100-specific IgG antibodies.
